# Metabolic analysis of the soil microbe *Dechloromonas aromatica *str. RCB: indications of a surprisingly complex life-style and cryptic anaerobic pathways for aromatic degradation

**DOI:** 10.1186/1471-2164-10-351

**Published:** 2009-08-03

**Authors:** Kennan Kellaris Salinero, Keith Keller, William S Feil, Helene Feil, Stephan Trong, Genevieve Di Bartolo, Alla Lapidus

**Affiliations:** 1 Department of Plant and Microbial Biology, University of California, Berkeley, CA 94720, USA; 2 Physical Biosciences Division, Lawrence Berkeley National Laboratories, Berkeley, CA 94710, USA; 3 Genomics Division, DOE Joint Genome Institute, Walnut Creek, CA 94598, USA; 4 Yámana Science and Technology, Washington, DC 20009, USA; 5 Land for Urban Wildlife, Concord, CA 94527, USA; 6 Boards 'N More, Brentwood, CA 94513, USA

## Abstract

**Background:**

Initial interest in *Dechloromonas aromatica *strain RCB arose from its ability to anaerobically degrade benzene. It is also able to reduce perchlorate and oxidize chlorobenzoate, toluene, and xylene, creating interest in using this organism for bioremediation. Little physiological data has been published for this microbe. It is considered to be a free-living organism.

**Results:**

The *a priori *prediction that the *D. aromatica *genome would contain previously characterized "central" enzymes to support anaerobic aromatic degradation of benzene proved to be false, suggesting the presence of novel anaerobic aromatic degradation pathways in this species. These missing pathways include the benzylsuccinate synthase (*bss*ABC) genes (responsible for fumarate addition to toluene) and the central benzoyl-CoA pathway for monoaromatics. In depth analyses using existing TIGRfam, COG, and InterPro models, and the creation of *de novo *HMM models, indicate a highly complex lifestyle with a large number of environmental sensors and signaling pathways, including a relatively large number of GGDEF domain signal receptors and multiple quorum sensors. A number of proteins indicate interactions with an as yet unknown host, as indicated by the presence of predicted cell host remodeling enzymes, effector enzymes, hemolysin-like proteins, adhesins, NO reductase, and both type III and type VI secretory complexes. Evidence of biofilm formation including a proposed exopolysaccharide complex and exosortase (epsH) are also present. Annotation described in this paper also reveals evidence for several metabolic pathways that have yet to be observed experimentally, including a sulphur oxidation (*sox*FCDYZAXB) gene cluster, Calvin cycle enzymes, and proteins involved in nitrogen fixation in other species (including RubisCo, ribulose-phosphate 3-epimerase, and nif gene families, respectively).

**Conclusion:**

Analysis of the *D. aromatica *genome indicates there is much to be learned regarding the metabolic capabilities, and life-style, for this microbial species. Examples of recent gene duplication events in signaling as well as dioxygenase clusters are present, indicating selective gene family expansion as a relatively recent event in *D. aromatica*'s evolutionary history. Gene families that constitute metabolic cycles presumed to create *D. aromatica'*s environmental 'foot-print' indicate a high level of diversification between its predicted capabilities and those of its close relatives, *A. aromaticum *str EbN1 and *Azoarcus *BH72.

## Background

*D. aromatica *strain RCB is a gram negative Betaproteobacterium found in soil environments [1]. Other members of the Betaproteobacteria class are found in environmental samples (such as soil and sludge) or are pathogens (such as *Ralstonia solanacearum *in plants and *Neisseria meningitidis *in humans) and in general the genus *Dechloromonas *has been found to be ubiquitous in the environment.

A facultative anaerobe, *D. aromatica *was initially isolated from Potomac River sludge contaminated with BTEX compounds (benzene, toluene, ethylbenzene and xylene) based on its ability to anaerobically degrade chlorobenzoate [1]. This microbe is capable of aromatic hydrocarbon degradation and perchlorate reduction, and can oxidize Fe(II) and H_2_S [2]. Although several members of the Rhodocyclales group of Betaproteobacteria are of interest to the scientific community due to their ability to anaerobically degrade derivatives of benzene, *D. aromatica *is the first pure culture capable of anaerobic degradation of the stable underivitized benzene molecule to be isolated. This, along with its ability to reduce perchlorate (a teratogenic contaminant introduced into the environment by man) and inquiry into its use in biocells [3] has led to interest in using this organism for bioremediation and energy production. Since the isolation of *D. aromatica*, other species of *Azoarcus *have been found to possess the ability to anaerobically degrade benzene, but have not been genomically sequenced [4].

The pathway for anaerobic benzene degradation has been partially deduced [5], but the enzymes responsible for this process have yet to be identified, and remain elusive even after the intensive annotation efforts described here-in. Conversely, central anaerobic pathways for aromatic compounds described in various other species were not found to be present in this genome [6].

## Methods

### Sequencing

Three libraries (3 kb, 8 kb and 30 kb) were generated by controlled shearing (Hydroshear, Genomic Solutions, Ann Arbor, MI) of spooled genomic DNA isolated from *D. aromatica* strain RCB and inserted into pUC18, pCUGIblu21, and pcc1Fos vectors, respectively. Clonal DNA was amplified using rolling circular amplification http://www.jgi.doe.gov/ and sequenced on ABI 3700 capillary DNA sequencers (Applied Biosystems, Foster City, CA) using BigDye technology (Perkin Elmer Corporation, Waltham, MA). Paired end-reads [7] were used to aid in assembly, and proved particularly useful in areas of repeats.

The Phrap algorithm [8,9] was used for initial assembly. Finishing and manual curation was conducted on CONSED v14 software [10], supplemented with a suite of finishing analysis tools provided by the Joint Genome Institute. *In silico *cross-over errors were corrected by manual creation of fake reads to guide the assembly by forcing the consensus to follow the correct path.

Gaps were closed through a combination of primer walks on the gap-spanning clones from the 3 and 8 kb libraries (identified by paired-end analysis in the CONSED software) as well as sequencing of mapped, unique PCR products from freshly prepared genomic DNA.

The final step required to create a finished single chromosomal sequence was to determine the number of tandem repeats for a 672 base DNA sequence of unknown length. This was done by creating the full tandem repeat insert from unique upstream and downstream primers using long-range PCR. We then determined the size of product (amplified DNA) between the unique sequences.

### Protein sequence predictions/orfs

Annotation done at Oak Ridge National Laboratory consisted of gene calls using CRITICA [11], glimmer [12], and Generation http://compbio.ornl.gov/. Annotation at the Virtual Institute for Microbial Stress and Survival http://www.microbesonline.org used bidirectional best hits as well as recruitment to TIGRfam hidden Markov models (HMMs), as described in Alm et al. [13]. Briefly, protein coding predictions derived from NCBI, or identified using CRITICA, with supplemental input from Glimmer, were analyzed for domain identities using the models deposited in the InterPro, UniProt, PRODOM, Pfam, PRINTS, SMART, PIR SuperFamily, SUPERFAMILY, and TIGRfam databases [13]. Orthologs were identified using bidirectional unique best hits with greater than 75% coverage. RPS-BLAST against the NCBI COGs (Clusters of Orthologous Genes) in the CDD database were used to assign proteins to COG models when the best hit E-value was <1e^-5 ^and coverage was >60%.

### Manual curation

Each and every predicted protein in the VIMSS database http://www.microbesonline.org[13] was assessed to compare insights obtained from recruitment to models from several databases (TIGRfams, COGs, EC and InterPro). Assignments that offered the most definitive functional assignment were captured in an excel spreadsheet with data entries for all proteins predicted in the VIMSS database. Extensive manual curation of the predicted protein set was carried out using a combination of tools including the VIMSS analysis tools, creation and assessment of HMMs, and phylogenomic analysis, as described [see Additional files 1, 2]. Changes in gene functional predictions and naming were captured in the excel spreadsheet, and predictions with strong phylogenetic evidence of function posted using the interactive VIMSS web-based annotation interface.

### Phylogenomic analysis: Flower Power, SCI PHY and HMM scoring

Hidden Markov models were generated for a large subset of proteins of interest, as detailed [see Additional file 1], to predict functional classification with the highest confidence measures currently available. The HMMs allowed recruitment of proteins to phylogenetic tree alignments that most closely reflect evolutionary relatedness across species. The proteins were assembled within clades of proteins that are aligned along their full length (no missing functional domains), and that allow high confidence of shared function in each species.

### Gene Family Expansion

A clustered set of paralogs [see Additional files 1, 2] was used to search for recent gene duplication events. After an initial assessment of the VIMSS gene information/homolog data, candidate proteins were used as seed sequences for Flower Power and internal tree-viewing tools or SCI-PHY analyses. These two approaches employed neighbor-joining trees using the Scoredist correction setting in the Belvu alignment editor, or the SCI-PHY utility and tree viewer. In either case resulting phylogenomic tree builds were reviewed, and contiguous protein alignments of two or more proteins from *D. aromatica *were considered to be candidates for a gene duplication event, either in the *D. aromatica *genome or in a predecessor species.

### Resequencing to verify absence of plasmid structure

After finishing the *D. aromatica *genome, analysis of the annotated gene set revealed the notable absence of several anaerobic aromatic degradation pathways that were expected to be present, due to their presence in *A. aromaticum *EbN1 (an evolutionary near-neighbor, as determined by 16sRNA phylogeny). Because many catabolic pathways are encoded on plasmid DNA, we felt it was important to preclude this possibility. We re-isolated DNA from a clonal preparation of *D. aromatica *that experimentally supported anaerobic benzene degradation, using three different plasmid purification protocols, each based on different physical parameters. All three generated a single band of DNA. The protocol that generated the highest yield of DNA was used to create a complete, new library of 2 kb inserts, and the library was submitted to sequence analysis using the protocols previously cited.

## Results

### Overview of Gene and Protein Features

The finished sequence for *D. aromatica *reveals a single circular, closed chromosome of 4,501,104 nucleotides created from 130,636 screened reads, with an average G+C content of 60% and an extremely high level of sequence coverage (average depth of 24 reads/base [see Additional file 3]). Specific probing for plasmids confirmed no plasmid structure was present in the clonal species sequenced, which supports anaerobic benzene degradation. It is noted however that the presence of two *tra *clusters (putative conjugal transfer genes; VIMSS582582-582597 and VIMSS582865-582880), as well as plasmid partitioning proteins, indicates this microbial species is likely to be transformationally competent and thus likely to be able to support plasmid DNA structures.

The Virtual Institute for Microbial Stress and Survival (VIMSS, http://www.microbesonline.org) and the Joint Genome Institute http://genome.jgi-psf.org/finished_microbes/decar/decar.home.html report 4170 and 4204 protein coding genes, respectively [see Additional file 3]. Cross-database comparisons were done to assure the highest probability of capturing candidate orfs for analysis. The majority of proteins are shared between data sets. Variations in N-termini start sites were noted, both between JGI and VIMSS datasets and between initial and later annotation runs (approximately 200 N-termini differences between four runs of orf predictions were noted for the initial two annotation runs, Joint Genome Institute's, done at Oak Ridge National Laboratories – ORNL, and VIMSS).

The most definitive functional classification, TIGRfams, initially defined approximately 10% of the proteins in this genome; as of this writing, 33% of predicted proteins in the *D. aromatica *genome are covered by TIGRfams, leaving 2802 genes with no TIGRfam classification [see Additional file 4]. Many proteins in the current and initial non-covered sets were investigated further using K. Sjölander's HMM building protocols (many of which are available at http://phylogenomics.berkeley.edu), to supplement TIGRfams. The Clusters of Orthologous Genes (COG) assignments were used for classification in the families of signaling proteins, but specific function predictions for these proteins also required further analyses. The metabolic and signaling pathways are discussed below, and the identity of orthologs within these pathways are based on analysis of phylogenomic profiles of clusters obtained by HMM analysis, with comparison to proteins having experimentally defined function.

### Anaerobic aromatic degradation – absence of known enzymes indicates novel pathways

One of the more striking findings is the absence of known key enzymes for monoaromatic degradation under anaerobic conditions. One of the primary metabolic capabilities of interest for this microbe is anaerobic degradation of benzene. Fumarate addition to toluene via benzylsuccinate synthase (BssABCD) is recognized as the common mechanism for anaerobic degradation by a phylogenomically diverse population of microbes [14-16] and has been called "the paradigm of anaerobic hydrocarbon oxidation"[17]. Benzoyl CoA is likewise considered a central intermediate in anaerobic degradation, and is further catabolized via benzoyl CoA reductase (BcrAB) [17]. Populated KEGG maps in the IMG and VIMSS databases, based on BLAST analyses, indicate the presence of some of the enzymes previously characterized as belonging to the Bss pathway in *D. aromatica*, yet more careful analysis shows the candidate enzymes to be members of a general family, rather than true orthologs of the enzymes in question. The majority of catabolic enzymes of interest for *D. aromatica *are not covered by TIGRfams or COGs families. For this reason Flower Power clustering, SCI-PHY subfamily clade analysis, and HMM scoring were used to ascertain the presence or absence of proteins of interest (for a detailed description, see Additional file 1). The most reliable prediction-of-function approaches for genomically sequenced protein orfs are obtained using the more computationally intensive HMM modelling and scoring utilities. This allows the protein in question to be assessed by phylogenetic alignment to protein families or sub-families with experimentally known function, providing much more accurate predictions [18,19].

To explore the apparent lack of anaerobic aromatic degradation pathways expected to be present in this genome, all characterized anaerobic aromatic degradation pathways from *A. aromaticum *EbN1 [20] were defined by HMMs to establish presence or absence of proteins in both the *D. aromatica *and *Azoarcus *BH72 genomes (these three genomes comprise nearest-neighbor species in currently sequenced species [see Additional file 1]. In *A. aromaticum *EbN1, ten major catabolic pathways have been found for anaerobic aromatic degradation, and nine of the ten converge on benzoyl-CoA [21]. A key catalytic enzyme or subunit for each enzymatic step was used as a seed sequence to recruit proteins from a non-redundant set of Genbank proteins for phylogenetic analysis. Benzylsuccinate synthase, present in *A. aromaticum *EbN1 [20,22] as well as *Thauera aromatica *[6], and *Geobacter metallireducens *[23], is not present in either the *D. aromatica *or *Azoarcus *BH72 genomes (see Table 1). Benzoyl-CoA reductase and benzylsuccinate synthase, previously denoted as "central" to anaerobic catabolism of aromatics, are likewise absent. The set of recruited proteins for both benzylsuccinate synthase and benzoyl-CoA reductase indicate they are not as universally present as has been suggested. *D. aromatica *does encode a protein in the pyruvate formate lyase family, but further analysis shows that it is more closely related to the *E. coli *homolog of this protein (which is not involved in aromatic catabolism) than to BssA. Anaerobic reduction of ethylbenzene is carried out by ethylbenzene dehydrogenase (EbdABCD1, 2) in *A. aromaticum*. This complex belongs to the membrane bound nitrate reductase (NarDKGHJI) family. In *D. aromatica*, this complex of proteins is only present as the enzymatically characterized perchlorate reductase (PcrABCD; [24]) which utilizes perchlorate, rather than nitrate, as the electron acceptor. EbdABCD proteins in *A. aromaticum *(VIMSS814904-814907 and VIMSS816928-816931) occur in operons that include (S)-1-phenylethanol dehydrogenases (Ped; VIMSS 814903 and 816927) [25], both of which are absent from *D. aromatica*, as is the acetophenone carboxylase that catalyzes ATP-dependent carboxylation of acetophonenone produced by Ped.

**Table 1:** Anaerobic aromatic degradation enzymes in near-neighbor *Aromatoleum aromaticum *EbN1.

Proteins involved in the anaerobic aromatic pathways in *Aromatoleum aromaticum *str. EbN1	*A. aromaticum *EbN1 – representative protein used for HMM models	*Azoarcus *BH72 ortholog	*D. aromatica *RCB ortholog
1) phenylalanine			
Pat	VIMSS813888:pat (COG1448; EC 2.6.1.57)	-	-
Pdc	VIMSS817385:pdc (COG3961)	-	-
Pdh	VIMSS816687:pdh (COG1012)	-	-
IorAB	VIMSS813644:iorA (COG4321)	-	+

2) phenylacetate			
PadBCD	VIMSS816693:padB	-	-
PadEFGHI	VIMSS816700:padI	-	-
PadJ	VIMSS816701:padJ	-	-

3) benzyl alcohol/benzaldehyde			
Adh	VIMSS815388:adh (COG1062)	-	-
Ald	VIMSS816847:ald (COG1012; EC1.2.1.28)	+	-

4) p-cresol			
PchCF	VIMSS813733:pchC (EC: 1.17.99.1)	-	-
PchA	VIMSS815385:pchC	-	-
	VIMSS813734:pchF (EC 1.1.3.38)	-	-
	VIMSS815387:pchF	-	-
	VIMSS815384:pchA (COG1012)	-	-

5) phenol			
PpsABC	VIMSS816923:ppsA phenylphosphate synthase	-	-
PpcABCD	VIMSS815367:ppcA	-	-

6) 4-hydroxybenzoate			
PcaK	VIMSS816471:pcaK (COG2271)	-	-
HbcL	VIMSS816681:hbcL1 4-hydroxybenzoate CoA ligase	-	-
HcrCBA	VIMSS815644:hcrB	-	-
	VIMSS815645:hcrA	-	-

7) toluene			
BssDCABEFGH	VIMSS814633:bssA	-	-
BbsABCDEFGH(IJ)	VIMSS814644:bbsH	-	-
	VIMSS814645:bbsG	-	-
	VIMSS814647:bbsF	-	-
	VIMSS814649:bbsD	-	-
	VIMSS814651:bbsB	-	-

8) ethylbenzene			
EbdABC	VIMSS814907:ebdA	-	+ (PcrA)
Ped	VIMSS814906:ebdB	-	+ (PcrB)
	VIMSS814905:ebdC	-	+ (PcrC)
	VIMSS814904:ebdD	-	+ (PcrD)
	VIMSS814903:ped	-	-

9) benzoate			
BenK	VIMSS816652:benK	-	-
BclA	VIMSS815152:bclA	+	-
BcrCBAD	VIMSS813961:bcrB	-	-
Dch Had Oah	VIMSS813959:bcrA	-	-

10) 3-Hydroxybenzoate			
HbcL	VIMSS813951:hbcL 3-hydroxybenzoate CoA ligase	-	-
BcrADB'C'			

Anaerobic aromatic degradation enzymes in near-neighbor *Aromatoleum aromaticum *EbN1 are largely absent from *Azoarcus *BH72 and *D. aromatica *RCB. Protein profiles (HMMs) were used to detect the presence or absence of anaerobic enzymes involved in degradation of aromatic compounds in *Aromatoleum aromaticum*. The last two columns denote presence (+) or absence (-) of homologs in either the *Azoarcus *BH72 or the *D. aromatica *RCB genome of the proteins listed that are present in the *A. aromaticum *EbN1 genome. The ethylbenzene dehydrogenase molybdenum-containing enzyme complex (ebdABCD) is described by TIGRfams 3479, 3478 and 3482, which define a type II DMSO reductase family of enzymes that includes *D. aromatica*'s perchlorate reductase PcrABCD subunits.

For all pathways except the ubiquitous phenylacetic acid catabolic cluster, which is involved in the aerobic degradation of phenylalanine, and the PpcAB phenylphosphate carboxylase enzymes involved in phenol degradation via 4-hydroxybenzoate, all key anaerobic aromatic degradation proteins present in *A. aromaticum *EbN1 are missing from the *D. aromatica *genome (Table 1), and the majority are also not present in *Azoarcus *BH72. The lack of overlap for genes encoding anaerobic aromatic enzymes between these two species was completely unexpected, as both *A. aromaticum *EbN1 and *D. aromatica *are metabolically diverse degraders of aromatic compounds. In general *Azoarcus *BH72 appears to share many families of proteins with *D. aromatica *that are not present in *A. aromaticum *EbN1 (eg signaling proteins, noted below).

Anaerobic degradation of benzene occurs at relatively sluggish reaction rates, indicating that the pathways incumbent in *D. aromatica *for aromatic degradation under anaerobic conditions might serve in a detoxification role. Another intriguing possibility is that oxidation is dependent on intracellularly produced oxygen, which is likely to be a rate-limiting step. *Alicycliphilus denitrificans *strain BC couples benzene degradation under anoxic conditions with chlorate reduction, utilizing the oxygen produced by chlorite dismutase in conjunction with a monooxygenase and subsequent catechol degradation for benzene catabolism [26]. A similar mechanism may account for anaerobic benzene oxidation coupled to perchlorate and chlorate reduction in *D. aromatica*. However, anaerobic benzene degradation coupled with nitrate reduction is also utilized by this organism, and remains enigmatic [5].

The extremely high divergence of encoded protein families in this functional grouping differs from the general population of central metabolic and housekeeping genes: *Azoarcus *BH72, *Azoarcus aromaticum *EbN1 and *D. aromatica *are evolutionarily near-neighbors within currently sequenced genomes, as defined both by the high level of protein similarity within house-keeping genes (defined by the COG J family of proteins), and 16sRNA sequence. *Azoarcus *BH72 and *A. aromaticum *EbN1 display the highest percent similarity between housekeeping proteins within this triad, with 138 of the 156 COG J proteins in *A. aromaticum *EbN1 displaying highest similarity to their BH72 counterparts. On average these two genomes display 83.5% amino acid identity across shared COG J proteins. *D. aromatica *is an outlier in the triad, with higher similarity to *Azoarcus *BH72 than *A. aromaticum *EbN1 (43 of *D. aromatica*'s 169 COG J proteins are most homologous to *A. aromaticum *EbN1 orthologs with an average 71% identity, and 67 are most homologous to *Azoarcus *BH72 with an average 72% identity).

Comparative genomics have previously established that large amounts of DNA present in one species can be absent even from a different strain within the same species [27]. In addition, the underestimation of the diversity of aromatic catabolic pathways (both aerobic and anaerobic) has been noted previously [28], and a high level of enzymatic diversity has been seen for pathways that have the same starting and end products, including anaerobic benzoate oxidation [29].

### Aerobic aromatic degradation

**Figure 1:**
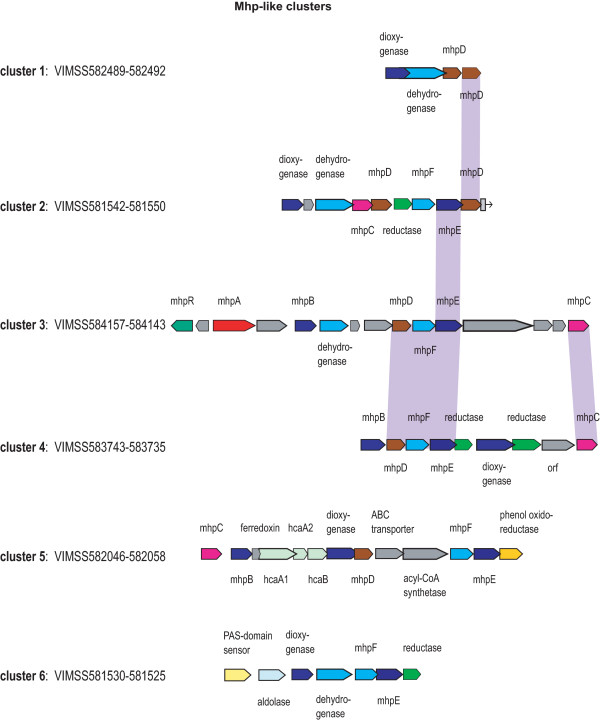
**Aerobic degradation of aromatic compounds: multiple Mhp-like dioxygenase clusters**. Each of the six *mhp*-like gene clusters in the *D. aromatica *genome is depicted. Recent gene duplications between individual proteins are shown by a purple connector between duplicates. Naming convention was chosen for simplicity and consistency, and names all proteins paralogous to a given Mhp protein with the Mhp name (MhpABCDEF or R), but does not imply enzymatic specificity for the substrates listed here-in, though the general enzymatic reaction is highly likely to be conserved. Mhp: *meta *cleavage of hpp, (hydroxyphenyl)propionate. MhpA, 3HPP hydroxylase; MhpB, DHPP 1,2-dioxygenase; MhpC, 2-hydroxy-6-ketonona-2,4-dienedioate hydrolase; MhpD, 2, deto-4-pentenoate hydratase; MhpE, 4-hydroxy-2-ketovalerate aldolas; MhpF, acetaldehyde dehydrogenase.

*D. aromatica *encodes several aerobic pathways for aromatic degradation, including six groups of oxygenase clusters that each share a high degree of sequence similarity to the phenylpropionate and phenol degradation (Hpp and Mhp) pathways in *Comamonas *species [30,31]. The *mhp *genes of *E. coli *and *Comamonas *are involved in catechol and protocatechuate pathways for aromatic degradation via hydroxylation, oxidation, and subsequent ring cleavage of the dioxygenated species. Only one of the clusters in *D. aromatica *encodes an *mhpA*-like gene; it begins with VIMSS584143 MhpC, and is composed of orthologs of MhpABCDEF&R, and is in the same overall order and orientation as the *Comamonas *cluster as well as the *E. coli mhp *gene families [32] (see Fig. 1, cluster 3). These pathways are also phylogenomically related to the biphenyl/polychlorinated biphenyl (Bhp) degradation pathways in *Pseudomonad *species [32]. For *Comamonas testosteroni*, this pathway is thought to be associated with lignin degradation [31]. Hydroxyphenyl propionate (HPP), an alkanoic acid of phenol, is the substrate for Mhp, and is also produced by animals in the digestive breakdown of polyphenols found in seed components [33]. Each gene cluster appears to represent a multi-component pathway, and is made up of five or more of various combinations of dioxygenase, hydroxylase, aldolase, dehydrogenase, hydratase, decarboxylase and thioesterase enzymes.

The single predicted MhpA protein in *D. aromatica *(VIMSS584155), which is predicted to support an initial hydroxylation of a substituted phenol substrate, shares 64.4% identity to *Rhodococcus *OhpB 3-(2-hydroxyphenyl) propionate monooxygenase (GI:8926385) vs. 26.4% for *Comamonas testosteroni *(GI:5689247), yet the remainder of the *ohp *genes in the *Rodococcus ohp *clade do not share synteny with the *D. aromatica mhp *gene cluster.

### Other aromatic oxygenases

**Figure 2:**
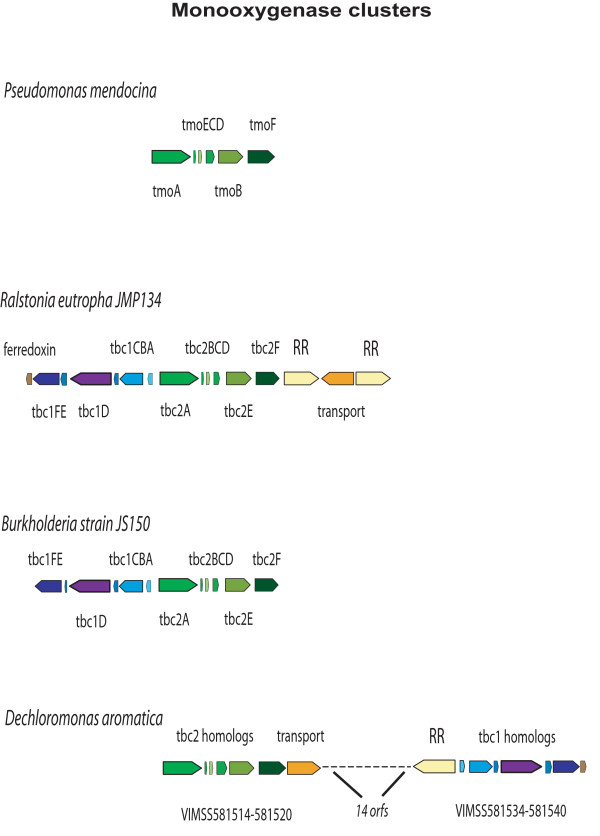
**Catabolic oxygenases of aromatic compounds: Synteny between *D. aromatica, P. mendocina, Burkholderia *and *R. eutropha***. Orthologous gene clusters for *P. mendocina, R. eutropha *JMP134, *Burkholderia *JS150 and *D. aromatica *are shown. *D. aromatica *possesses two oxygenase gene clusters that are syntenic to the *tbc*1 and 2 catabolic gene clusters of * Burkholderia *JS150, but with an inversion and insertion in the chromosome. Also shown are the *tmo *(toluene mono-oxygenase) toluene degradative cluster of *P. mendocina *and the *tbc*1 &* tbc*2-like (tcb: toluene, chlorobenzene, and benzene utilization) gene cluster of *R. eutropha *(VIMSS 896207–896222, *Burkholderia *protein names were used for consistency). The first seven orfs (encoding a *tbc*1-like cluster) of *R. eutropha *JMP134 are orthologous to the PoxABCDEFG (phenol hydroxylase) and P0123456 genes of *Ralstonia *sp E2 and *R. eutropha *H16, respectively. Orthologs can be identified as having the same size and color scheme.

Two chromosomally adjacent monooxygenase clusters, syntenic to genes found in *Burkholderia *and *Ralstonia *spp, indicate that *D. aromatica *might have broad substrate hydroxylases that support the degradation of toluene, vinyl chlorides, and TCE (Fig. 2 and Table 2), and are thus candidates for benzene-activating enzymes in the presence of oxygen.

**Table 2:** Aromatic degradation in *D. aromatica*: Mono- and Di-oxygenases.

VIMSS id	Orthologs	Putative function	Size aas
581514	TbuA1/TmoA/TouA/PhlK/Tbc2A	methane/phenol/toluene hydroxylase	501
581515	TbuU/TmoE/TouB/PhlL/Tbc2B	toluene-4-monooxygenase	88
581516	TbuB/TmoC/TouC/PhlM/Tbc2C	ferredoxin subunit of ring-hydroxylating dioxygenase	111
581517	TbuV/TmoD/TouD/PhlN/Tbc2C	monooxygenase	146
581518	TbuA2/TmoB/TouE/PhlO/Tbc2E	hydroxylase	328
581519	TbuC/TmoF/TouF/PhlP/Tbc2F	flavodoxin reductase	338
581520	TbuX/TodX/XylN	membrane protein; transport	464
581521	histidine kinase	signal transduction	963
581522	NarL	cheY like protein	208
581523	methyl-accepting chemotaxis protein	chemotaxis sensory transducer, membrane bound	532
581524	4-oxalocrotonate tautomerase	tautomerase	144
581525	oxidoreductase	oxidoreductase/dehydrogenase	254
581526	MhpE	4-hydroxy-2-oxovalerate aldolase	354
581527	MhpF	EC1.2.1.10 Acetaldehyde dehydrogenase (acetylating)	305
581528	2-hydroxymuconic semialdehyde dehydrogenase	NAD+-dependent dehydrogenase (EC1.2.1.60)	489
581529	ring-cleaving extradiol dioxygenase	catechol 2,3 dioxygenase (1.13.11.2)	311
581530	aldolase	4-hydroxyphenylacetic acid catabolism pathway	266
581531	S box domain	signal transduction	143
584293	orf	unknown	63
581532	orf	unknown	80
584294	EAL domain containing protein (obsolete in current VIMSS database)	diguanylate phosphodiesterase; signaling	65
581533	transcriptional regulator	LysR-type	300
581534	response regulator, tbuT family	activator of aromatic catabolism	558
812947	PhcK/DmpK/PhhK/PheA1/Tcb1A/AphK	monooxygenase	89
581535	PhcL/DmpL/PhhL/PheA2/Tcb1B/AphL	hydroxylase	329
581536	PhcM/DmpM/PhhM/PheA3/Tcb1C/AphM	monooxygenase	89
581537	PhcN/DmpN/PhhN/PheA4/Tcb1D	aromatic hydroxylase	517
581538	PhcO/DmpO/PhhO/PheA5/Tcb1E/AphO	aromatic hydroxylase	118
581539	PhcP/Dmp/PhhP/PheA6/Tcb1F/AphQ	hydroxylase reductase	353
581540	ferredoxin	2Fe-2S ferredoxin, iron-sulfur binding site	112
581541	transcriptional regulator	IPR000524: Bacterial regulatory protein GntR, HTH	235
581542	ring-cleaving extradiol dioxygenase	catechol 2,3 dioxygenase (EC1.13.11.2)	308
581543	orf	unknown	142
581544	2-hydroxymuconic semialdehyde dehydrogenase	NAD+-dependent dehydrogenase (EC1.2.1.60)	484
581545	MhpC	2-hydroxy-6-ketonona-2,4-dienedioic acid hydrolase	274
581546/3337834	MhpD	2-keto-4-pentenoate hydratase	260
581547	oxidoreductase	3-oxoacyl-[acyl-carrier-protein] reductase (EC1.1.1.100)	264
581548	MhpF	acetaldehyde dehydrogenase (acetylating; EC1.2.1.10)	304
581549	MhpE	4-hydroxy-2-oxovalerate aldolase	343
581550	hydratase/decarboxylase	4-oxalocrotonate decarboxylase	262
581551	tautomerase	4-oxalocrotonate tautomerase	63

The large cluster of aromatic degradation enzymes in the *D. aromatica *genome shown includes two mono-oxygenase clusters in a linear array on the *D. aromatica *chromosome, with 17 predicted genes intergenically inserted, which encode *mhp *'cluster 6' and several predicted signaling proteins. The second monooxygenase cluster is followed by *mhp *'cluster 2' (for an overview of *mhp *clusters see Fig. 1).

One monooxygenase gene cluster, composed of VIMSS581514 to 581519 ('tbc2 homologs,' Fig. 2), is orthologous to the *tbuA1UBVA2C*/*tmoAECDBF/touABCDEF/phlKLMNOP *and *tbc2ABCDEF *gene families (from *P. stutzeri, R. pickettii*, and *Burkholderia *JS150). This gene cluster includes a transport protein that is orthologous to TbuX/TodX/XylN (VIMSS581520). Specificity for the initial monooxygenase is not established, but phylogenetic analysis places VIMSS581514 monooxygenase with near-neighbors TbhA [34], reported as a toluene and aliphatic carbohydrate monooxygenase (76.5% sequence identity), and BmoA [35], a benzene monooxygenase of low regiospecificity (79.6% sequence identity). The high level of similarity to the *D. aromatica *protein is notable. The region is also highly syntenic with, and homologous to, the *tmo*AECDBF (AY552601) gene cluster responsible for *P. mendocina*'s ability to utilize toluene as a sole carbon and energy source [36].

Just downstream on the chromosome is a *phc/dmp/phh/phe/aph*-like cluster of genes, composed of the genes VIMSS812947 and VIMSS 581535 to 581540 ('tbc1 homologs,' Fig. 2). Overall, chromosomal organization is somewhat different for *D. aromatica *as compared to *Ralstonia *and *Burkholderia*. *D. aromatica *has a fourteen gene insert that encodes members of the *mhp*-like family of aromatic oxygenases between the tandem tbc 1 and 2-like oxygenase clusters (see Table 2), with an inversion of the second region compared to *R. eutropha *and *Burkholderia*. Clade analysis indicates a broad substrate phenol degradation pathway in this cluster, with high sequence identity to the TOM gene cluster of *Bradyrhizobium*, which has the ability to oxidize dichloroethylene, vinyl chlorides, and TCE [37,38]. The VIMSS581522 response regulator gene that occurs between the two identified monooxygenase gene clusters shares 50.3% identity to the *Thaurea aromatica tutB *gene and 48.2% to the *Pseudomonas *sp. Y2 styrene response regulator (occupying the same clade in phylogenetic analysis). VIMSS581522 is likely to be involved in the chemotactic response in conjunction with VIMSS581521 (histidine kinase) and VIMSS581523 (methyl accepting chemotaxis protein), which would confer the ability to display a chemotactic response to aromatic compounds.

Overall, several mono- and di-oxygenases were found in the genome, indicating *D. aromatica *has diverse abilities in the aerobic oxidation of heterocyclic compounds.

There are several gene clusters indicative of benzoate transport and catabolism. All recognized pathways are aerobic. The benzoate dioxygenase cluster BenABCDR is encoded in VIMSS582483-582487, and is very similar to (and clades with) the xylene degradation (*xyl*XYZ) cluster of *Pseudomonas*.

There is also an *hcaA *oxygenase gene cluster, embedded in one of the *mhp *clusters (see cluster 5, Fig. 1). Specificity of the large subunit of the dioxygenase (VIMSS582049) appears to be most likely for a bicyclic aromatic compound, as it shows highest identity to dibenzothiophene and naphthalene dioxygenases.

### *Dechloromonas aromatica's* sensitivity to the environment

#### Cell Signaling

*D. aromatica *has a large number of genes involved in signaling pathways, with 314 predicted signaling proteins categorized in COG T (signal transduction mechanisms) and a total of 395 proteins (nearly 10% of the genome) either recruited to COG T or possessing annotated signal transduction domains. Signaling appears to be an area that has undergone recent gene expansion, as nine recent gene duplication events in this functional group are predicted by phylogenetic analysis, as described in a later section (shown in Table 3).

**Table 3:** Candidates for gene expansion in the *D. aromatica *genome.

Protein/protein family function	Number of duplicates	Number of triplicates
Transport (membrane)	12	
Signal transduction or regulatory – includes:	9	
FlhD homolog	(1)	
FlhC homolog	(1)	
Nitrogen regulatory protein PII homolog	(1)	
Hydrolase/transhydrogenase or hydratase	4	1
Cytochromes	3	2
Mhp family	2	2
Phospholipase/phosphohydrolase	2	1
Phasin	1	
Dioxygenase	1	
NapH homolog	1	
NosZ homolog	1	
Unknown function	7	

Proteins within the genome that show evidence of possible recent gene duplication are tabulated by general functional group, or, in some cases, specific proteins (NapH, NosZ, FlhCD, Nitrogen regulatory protein PII). Duplicates and triplicates were determined by adjacent clustering of the *D. aromatica *proteins in a phylogenomic tree profile. The percent identity between the *D. aromatica *duplicate and triplicate candidates is higher than identity to other species' protein candidates, indicating a possible gene family expansion event. Areas of duplicated clusters of proteins (for instance, the regions surrounding VIMSS582581, 582612, 582641, 582657, 582863, 583914 and 583592), including phage elements and Tra-type conjugation proteins, are not included in this table. Parentheses indicate these duplication events have been tabulated in the general category of signal transduction or regulatory proteins – individual protein types of particular interest are noted separately by protein name.

**Figure 3:**
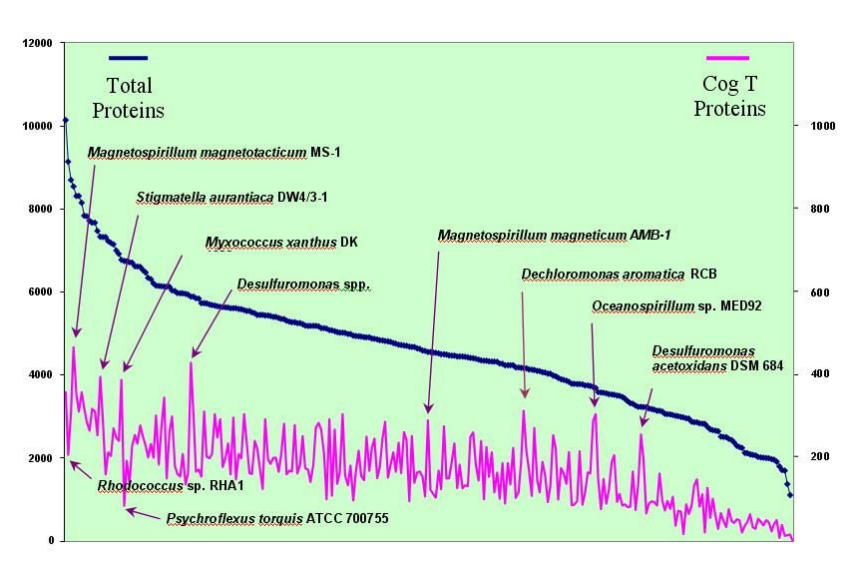
**Number of predicted signaling proteins versus total protein count**. Microbial genomes, displaying total number of predicted open reading frames (orfs, left axis) and total number of predicted signaling proteins (defined as COG T, right axis). Microbes displaying a high number of signaling orfs relative to total predicted proteins are labelled (above COG T line), as well as two large-sized genomes having a relatively low number of annotated COG T proteins (labelled below COG T line).

**Figure 4:**
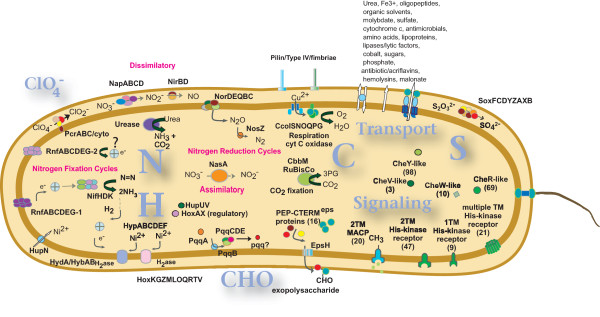
**Overview of predicted metabolic cycles, membrane transporters and signaling proteins in *D. aromatica***. Various metabolic cycles, secretory apparatus and signaling cascades predicted in the annotation process are depicted. TM: transmembrane. Gene names are discussed in the relevant sections of this paper. Areas of the cell depicting Nitrogen, Hydrogen, Carbon and Sulfur cycles are indicated by "N," "H," "C," and "S."

Complex lifestyles are implicated in large genomes with diverse signaling capability, and in general genomes with a very large number of annotated open reading frames (orfs) have high numbers of predicted signal transducing proteins, as shown in Fig. 3, though some species, such as *Rhodococcus *RHA1 and *Psychroflexus torques *are notable exceptions to this trend. However, assessment of COG T population size relative to other genomes with a similar number of predicted orfs (Fig. 3) indicates that *D. aromatica *is one of a handful of species that have a large relative number of signaling proteins vs similarly sized genomes. Other organisms displaying this characteristic include *Magnetospirillum magnetotacticum *MS-1, *Stigmatella aurantiaca, Myxococcus Xanthus *DK1622, *Magnetospirillum magneticum *AMB-1, *Oceanospirillum sp*. MED92, and *Desulfuromonas acetoxidans*. Within the Betaproteobacteria, *Chromobacterium violaceum *and *Thiobacillus denitrificans *have a relatively large number of signaling cascade genes, but still have far fewer than found in *D. aromatica*, with 262 predicted COG T proteins (6% of the genome) and 137 COG T proteins (4.8% of the genome), respectively. Histidine kinase encoding proteins are particularly well-represented, with only *Stigmatella aurantiaca *DW4/3-1, *Magnetococcus *sp. MC-1, *Myxococcus xanthus *DK 1622, and *Nostoc punctiforme *reported as having more. The sixty-eight annotated histidine kinases include a large number of nitrate/nitrogen responsive elements. Furthermore, the presence of 47 putative histidine kinases predicted to contain two transmembrane (TM) domains, likely to encode membrane-bound sensors (see Fig. 4), suggests that *D. aromatica *is likely to be highly sensitive to environmental signals. Nearly half (48%) of the predicted histidine kinases are contiguous to a putative response regulator on the chromosomal DNA, indicating they likely constitute functionally expressed kinase/response regulator pairs. This is atypically high for contiguous placement on the chromosome [39].

A relatively high level of diguanylate cyclase (GGDEF domain [40-42]) signaling capability is implied in *D. aromatica *by the presence of 57 proteins encoding a GGDEF domain (Interpro IPR000160 [see Additional file 5]) and an additional 10 with a GGDEF response regulator (COG1639) [40]. *E. coli*, for comparison, encodes 19. This gene family also appears to have undergone recent expansion in this microbe's evolutionary history. Microbes having a large number of proteins or even a diverse array of COG T elements do not *a priori *encode a large number of GGDEF elements, as *Stigmatella aurantiaca*, *Myxococcus, Xanthus *DK1622 and *Burkholderia pseudomallei*, by contrast, have very large genomes with extensive COG T populations, yet each have 20 or fewer proteins identified as having GGDEF domains [see Additional file 5], and *Prochloroccus *spp. appear to have none. Conversely, *Oceanospirillum *has a relatively small genome, yet has 112 proteins identified as likely GGDEF domain/IPR000160 proteins. GGDEF/EAL domain response regulators have been implicated in root colonization in *Pseudomonas putida *(Matilla et al. 2007); in *E. coli *the GGDEF domain-containing YddV protein upregulates the transcription of a number of cell wall modification enzymes [42], and in point of fact, *D. aromatica*'s VIMSS581804, a GGDEF domain containing homolog of the YddV *E. coli *protein, occurs upstream of a cluster of sixteen cell wall division proteins (encoded by VIMSS581805-581820).

### Cellular interactions with community/environment – secretion

#### TypeI secretion

Fifteen transport clusters include a TolC-like outer membrane component, and recent gene family expansion is noted within several families of ABC transporters for this genome. TolC was originally identified in *E. coli *as the channel that exports hemolysin [43], and hemolysin-like proteins are encoded in this genome. Two groups of ABC transporters occur as a cluster of five transport genes; these five-component transporters have been implicated in the uptake of external macromolecules [44].

The presence of putative lytic factors, lipases, proteases, antimicrobials, invasins, hemolysins, RTXs and colicins near potential type I transport systems indicate that these might be effector molecules used by *D. aromatica *for interactions with host cells (eg. for cell wall remodeling). Iron acquisition is likely to be supported by a putative FeoAB protein cluster (VIMSS583997, 583998), as well as several siderophore-like receptors and a putative FhuE protein (outer membrane receptor for ferric iron uptake; VIMSS583312). Other effector-type proteins, likely to be involved in cell/host interactions (and which in some species have a role in pathogenicity [45]), are present in this genome. Adhesins, haemagglutinins, and oxidative stress neutralizers are relatively abundant in *D. aromatica*. A number of transporters occur near the six putative soluble lytic murein transglycosylases, indicating possible cell wall remodeling capabilities for host colonization in conjunction with the potential effector molecules noted above. Homologs of these transporters were shown to support invasin-type functions in other microbes [45]. Interaction with a host is further implicated by: VIMSS581582, encoding a potential cell wall-associated hydrolase, VIMSS581622, encoding a predicted ATPase, and VIMSS3337824/formerly 581623, encoding a putative membrane-bound lytic transglycosylase.

Eleven tandem copies of a 672 nucleotide insert comprise a region of the chromosome that challenged the correct assembly of the genome, and finishing this region was the final step for the sequencing phase of this project (see Methods). Unexpectedly, analysis of this region revealed a potential open reading frame encoding a very large protein that has been variously predicted at 4854, 2519 or 2491 amino acids in size during sequential automated protein prediction analyses (VIMSS3337779/formerly 582095). This putative protein, even in its smallest configuration, contains a hemolysin-type calcium-binding region, a cadherin-like domain, and several RTX domains, which have been associated with adhesion and virulence. Internal repeats of up to 100 residues with multiple copies have also been found in proteins from *Vibrio, Colwellia, Bradyrhizobium*, and *Shewanella *spp. (termed "VCBS" proteins as defined by TIGRfam1965).

Other potential effector proteins include: three hemolysin-like proteins adjacent to type I transporters, eight proteins with a predicted hemolysin-related function, including VIMSS583067, a hemolysin activation/secretion protein, VIMSS580979, hemolysin A, VIMSS583372, phospholipase/hemolysin, VIMSS581868, a homolog of hemolysin III, predicted by TIGRfam1065 to have cytolytic capability, VIMSS582079, a transport/hemolysin, and VIMSS581408, a general hemolysin. Five predicted proteins have possible LysM/invasin domains, including: VIMSS580547, 581221, 581781, 582766, and 583769. One gene, VIMSS583068, encodes a putative 2079 amino acid filamentous haemagglutinin, as well as a hasA-like domain, making it a candidate for hasA-like function (hasA is a hemophore that captures heme for iron acquisition [46]).

#### Type II secretion

Besides the constitutive Sec and Tat pathways, *D. aromatica *has several candidates for dedicated export secretons of unknown function, with 3–4 putative orthologs of PulDEFG interspersed with a lytic transglycosylase and a hemolysin (VIMSS582071-582085). The region from VIMSS581889 to VIMSS581897 includes *pul*DEFG type subunits and an *exe*A ATPase like protein. It is bracketed by signaling components comprised of a histidine kinase, adenylate cyclase, and a protein bearing similarity to the nitrogen response regulator *gln*G (VIMSS581898), which has been shown to be involved in NH_3 _assimilation in other species [47].

In addition, there is a nine-gene cluster that encodes several proteins related to toluene resistance (VIMSS581899 to 581906).

A pilus-like gene cluster (which can also be classified as type IV secretion) occurs in VIMSS580547-580553, encoding a putative lytic transglycosylase, ABC permease, cation transporter, pilin peptidase, pilin ATPase and PulF-type protein. This assembly resembles other pilin assemblies associated with attachment to a substrate, such as the pilus structure responsible for chitin/host colonization in *Vibrio cholerae *[48].

Another large pilus-like cluster (VIMSS584160-584173) occurs in close proximity to the *mhp*CEFDBAR oxygenase genes (see eg VIMSS584157, *mhp*R).

#### Type III secretion

*D. aromatica *has been shown to be chemotactic under various circumstances. The flagellar proteins (FliAEFGHIJKLMNOPQR, FlaABCDEFGHIJK) are followed by an additional cluster of 15 chemotaxis/signal transduction genes (VIMSS580462-580476), and homologs of FlhC and D regulatory elements required for the expression of flagellar proteins (VIMSS582640 and 582641) [49], identified by phylogenetic clustering, are also present. Since *D. aromatica *has a flagellum and displays chemotactic behavior, it is likely that the flagellar gene cluster is solely related to locomotion, though type III secretion systems can also encode dedicated protein translocation machineries that deliver bacterial pathogenicity proteins directly to the cytosol of eukaryotic host cells [50].

#### Type IV secretion

There are two copies of a twenty-one gene cluster that includes ten putative conjugal transfer (Tra) sex-pilus type genes in the *D. aromatica *genome (VIMSS582582-582601 and VIMSS582864-582884), indicating a typeIV secretion structure that is related to non-pathogenic cell-cell interactions [51].

#### Type VI secretion

A large cluster of transport proteins that is related to the virulence associated genetic locus HIS-1 of *Pseudomonas aeruginosa *and the VAS genes of *V. cholerae *[52,53] includes homologs of hcp1, IcmF and clpV (as VIMSS583005, 582995 and 583009, respectively, in *D. aromatica *[see Additional file 6]). This IcmF-associated (IAHP) cluster has been associated with mediation of host interactions, via export of effector proteins that lack signal sequences [53]. Further evidence for type VI secretion is found in the presence of three proteins containing a Vgr secretion motif modeled by TIGRfam3361, which is found only in genomes having type VI secretory apparatus. Though most bacteria that contain IcmF clusters are pathogenic agents that associate with eukaryotic cell hosts [54], it has been reported that the host interactions supported by this cluster are not restricted to pathogens [55].

The type IV pili systems might be involved in biofilm development, as interactions with biofilm surfaces are affected by force-generating motility structures, including type IV pili and flagella [56]. Quorum sensing is a deciding input for biofilm formation, and the presence of an exopolysaccharide synthetic cluster lends further support for biofilm formation. Further, derivatives of nitrous oxide, which is an evident substrate for *D. aromatica*, are a key signal for biofilm formation vs cell dispersion in the microbe *P. aeruginosa *[57].

### Cellular interactions with community – quorum sensing

Quorum sensing uses specific membrane bound receptors to detect autoinducers released into the environment. It is involved in both intra- and inter-species density detection [58,59]. Cell density has been shown to regulate a number of cellular responses, including bioluminescence, swarming, expression of virulence factors, secretion, and motility (as reviewed in Withers et al. 2001 [60]).

*D. aromatica *encodes six histidine kinase receptor proteins that are similar to the quorum sensing protein QseC of *E. coli *(VIMSS580745, 582451, 582897, 583274, 3337577 (formerly 583538), and 583893), five of which co-occur on the chromosome with homologs of the CheY like QseB regulator, and two of which appear to be the product of a recent duplication event (VIMSS583893 & 3337577). Of the six QseC homologs, phylogenetic analysis indicates VIMSS582451 is most similar to QseC from *E. coli*, where the QseBC complex regulates motility via the FlhCD master flagellar regulators (VIMSS582640 and 582641). The presence of several *qseC/B *gene pairs indicates the possibility of specific responses that are dependant on different sensing strategies. In other species, expression of ABC exporters is regulated by quorum sensing systems [46]; gene family expansion is indicated in the ABC export gene pool as well as the qseC/B sensors in *D. aromatica*.

N-acyl-homoserine lactone is the autoinducer typical for gram negative bacteria [61], yet *D. aromatica *lacks any recognizable AHL synthesis genes. *Ralstonia *Betaproteobacteria likewise encode several proteins in the *qse*C gene family and display a diversity of candidate cell density signaling compounds other than AHL [62]. The utility of having a diverse array of quorum sensing proteins remains to be determined, but appears likely to be associated with a complex, and possibly symbiotic, lifestyle for *D. aromatica*.

### Cellular interactions with the environment – stress

#### Carbon Storage

Poly-hydroxyalkanoates (PHAs) store carbon energy, are synthesized from the catabolism of lipids, and constitute up to almost 90% of the dry weight of the Betaproteobacteria species *Comamonas testosteroni *[63]. These lipid-like carbon/energy storage polymers are found in granular inclusions. PhaR candidate VIMSS583509 is likely to be a regulatory protein for PHA synthesis, and is found near other proteins associated with PHA granule biosynthesis and utilization in *D. aromatica *(VIMSS583511-513).

Phasins are relatively small proteins (180–200 aas) that have been shown to associate with PHA inclusions [64]. There are six copies of phasin-type proteins, with indications of recent gene duplication for three of the phasin-type proteins (VIMSS581881, 582264, and 3337571 (formerly 583582)). There are also three homologs of the active subunit poly-B-hydroxybutyrate polymerase (PhaC orthologs) and two Pha reductase candidates present in a direct repeat, which is also found in *Legionella pneumophila*. Interestingly, one PhaC-like protein, VIMSS583511, is 70% identical to NodG of *Azospirillum brasilense*, a nodulation protein [65]. No PhaA-like ketothiolase ortholog is present. The presence of an amplified gene pool for carbon storage granules in *D. aromatica *may confer the ability to survive under low nutrient conditions, and poly-3-hydroxybutyrate accumulation has recently been observed in *A. aromaticum *EbN1 cultures displaying reduced growth [66].

#### Phosphate

Inorganic polyphosphate storage appears likely, as both polyphosphate kinase (Ppk, VIMSS582444) and exopolyphosphatase (Ppx, VIMSS583870) are present. These genes are similar to those encoded in *Pseudomonas aeruginosa*, in that they are in disparate regions of the chromosome [67]. Polyphosphate has been implicated in stress response due to low nutrients in the environment [68], and also in DNA uptake [69].

Phosphate transport appears to be encoded in a large cluster of genes (VIMSS581746-581752), and response to phosphate starvation is likely supported by the PhoH homolog VIMSS583854.

#### Biofilm formation

There is a large cluster of exopolysaccharide export (eps) associated genes, including a proposed exosortase (epsH, VIMSS582792). Presence of the eps family proteins (VIMSS582786, VIMSS582790-582801) indicates capsular exopolysaccharide production, associated with either host cell interactions (including root colonization [70]) or biofilm production in soil sediments [71]. *D. aromatica *is one of a small number of species (19 out of 280 genomes assessed by Haft et al. [71]) that also encodes the PEP-CTERM export system. The PEP-CTERM signal, present in sixteen proteins in this genome, is proposed to be exported via a potential exportase, represented in this genome by epsH (VIMSS582792). Additionally, the presence of proteins encoding this putative exportase is seen only in genomes also encoding the eps genes.

### Metabolic Cycles

#### Nitrogen

*D. aromatica *closely reflects several metabolic pathways of *R. capsulatus*, which is present in the rhizosphere, and its assimilatory nitrate/nitrite reductase cluster is highly similar to the *R. capsulatus *cluster [72]. Encoded nitrate response elements also indicate a possible plant association for this microbe, as nitrate can act as a terminal electron acceptor in the oxygen-limited rhizosphere. Alternatively, nitrous oxide (NO) reduction can indicate the ability to respond to anti-microbial NO production by a host (used by the host to mitigate infection [73]). Several gene families are present that indicate interactions with a eukaryotic host species, including response elements that potentially neutralize host defense molecules, in particular nitric oxide and other nitrogenous species.

Nitrate is imported into the cytosol by NasDEF in *Klebsiella pneumoniae *[74] and expression of nitrate and nitrite reductases is regulated by the nasT protein in *Azotobacter vinelandii *[75]. A homologous set of these genes are encoded by the cluster VIMSS580377-580380 (NasDEFT), and a homolog of *nar*K is immediately downstream at VIMSS580384, and is likely involved in nitrite extrusion. Upstream, a putative *nas*A/*nir*BDC cluster (assimilatory nitrate and nitrite reduction) is encoded near the *nar*XL-like nitrate response element. VIMSS580393 encodes a nitrate reductase that is homologous to the NasA cytosolic nitrate reductase of *Klebsiella pneumoniae *[76]. Community studies have correlated the presence of NasA-encoding bacteria with the ability to use nitrate as the sole source of nitrogen [77]. The large and small subunits of nitrite reductase (VIMSS580391 *nir*B and VIMSS580390 *nir*D) are immediately adjacent to a transporter with a putative nitrite transport function (VIMSS580389 NirC-like protein). The NirB orf is also highly homologous to *both *NasB (nitrite reductase) and NasC (NADH reductase which passes electrons to NasA) of *Klebsiella pneumoniae*. HMMs created from alignments seeded by the NasB and NasC genes scored at 3.2e^-193 ^and 4.0e^-159^, respectively, to the VIMSS580391 NirB protein. *D. aromatica *is similar to *Methylococcus capsulatus, Ralstonia solanacearum, Polaromonas*, and *Rhodoferax ferrireducens *for *nas*A, *nir*B and *nir*D gene clusters. However, the presence of the putative transporter *nir*C (VIMSS580389) shares unique similarity to the *E. coli *and *Salmonella nir*BCD clusters.

Putative periplasmic, dissimilatory nitrate reduction, which is a candidate for denitrification capability [78], is encoded by the *nap*DABC genes (VIMSS 3337807/581796-581799). A probable cytochrome c', implicated in nitric oxide binding as protection against potentially toxic excess NO generated during nitrite reduction [79], is encoded by VIMSS582015. Although most denitrifiers are free living, plant-associated denitrifiers do exist [80]. There is no dissimilatory nitrate reductive complex *nar*GHIJ, but rather, NarG and NarH-like proteins are found in the evolutionarily-related perchlorate reductase alpha and beta subunits [24]. These proteins are present in the *pcr*ABCD*cld *cluster, VIMSS582649-582652 and VIMSS584327, as previously reported for *Dechloromonas *species [81].

Ammonia incorporation appears to be metabolically feasible via a putative glu-ammonia ligase (VIMSS581081), an enzyme that incorporates free ammonia into the cell via ligation to a glutamic acid. An ammonium transporter and cognate regulator are likely encoded in the Amt and GlnK-like proteins VIMSS581101 and 581102.

Urea catabolism as a further source of nitrogen is suggested by two different urea degradation enzyme clusters. The first co-occurs with a urea ABC-transport system, just upstream of a putative nickel-dependent urea amidohydrolase (urease) enzyme cluster (VIMSS583666, 583671–583674, and VIMSS583677-583683; see Table 4). The second pathway is suggested by a cluster of urea carboxylase/allophanate hydrolase enzymes (VIMSS581083-581085, described by TIGRfams 1891, 2712, 2713, 3424 and 3425), which comprise four proteins involved in urea degradation to ammonia and carbon dioxide in other species, as well as an amidohydrolase [82].

**Table 4:** Putative nitrogen fixation gene cluster in *D. aromatica*

VIMSS id	Ortholog	Size, aas
583652	FldA, flavodoxin typical for nitrogen fixation	186
583653	hypothetical protein	86
583654	NafY-1, nitrogenase accessory factor Y	247
583655	NifB, nitrogenase cofactor biosynthesis protein	500
583656	4Fe-4S ferredoxin	92
583657	nitrogenase-associated protein	159
583658	flavodoxin	423
583659	ferredoxin, nitric oxide synthase	95
583660	2Fe-2S ferredoxin	120
583661	NifQ	190
583662	DraG	326
583663	histidine kinase	1131
583664	Che-Y like receiver	308
583666	UrtA urea transport	420
583667/3337562	CynS cyanate lyase	147
583668	S-box sensor, similar to oxygen sensor arcB	794
583669	ABC transporter	393
3337561	Protein of unknown function involved in nitrogen fixation	72
583671	UrtB urea transport	525
583672	UrtC urea transport	371
583673	UrtD urea transport	278
583674	UrtE urea transport	230
583677	UreH urease accessory protein	288
583678	Urea amidohydrolase gamma	100
583679	Urea amidohydrolase beta	101
583680	Urea amidohydrolase alpha/UreC urease accessory protein	569
583681	UreE urease accessory protein	175
583682	UreF urease accessory protein	228
583683	UreG urease accessory protein	201
583685	nitroreductase	558
583686	ferredoxin, subunit of nitrite reductase	122
583691	DraT	328
583692	NifH nitrogenase iron protein (EC1.18.6.1)	296
583693	NifD nitrogenase molybdenum-iron protein alpha chain (EC1.18.6.1)	490
583694	NifK nitrogenase molybdenum-iron protein beta chain (EC1.18.6.1)	522
583695/3337559	NifT	80
3337558	ferredoxin	63
583696	NafY-2 nitrogenase accessory factor Y	243
583710/3337556	NifW nitrogen fixation protein	113
3337555	NifZ	151
583711/3337554	NifM	271

The annotated nitrogen fixation homologs (Nif proteins) are embedded with a cluster of urea transport and degradation genes (Ure, Urea amidohydrolase and Urt transport families).

#### Nitric oxide (NO) reductase

The chromosomal region around *D. aromatica'*s two *nos*Z homologs is notably different from near-neighbors *A. aromaticum *EbN1 and *Ralstonia solanacearum *which encode a nosRZDFYL cluster. *D. aromatica*'s *nosRZDFYL *operon lacks the nosRFYL genes, and displays other notable differences with most nitrate reducing microbes. In *D. aromatica*, two identical *nos*Z reductase-like genes (annotated as *nos*Z1 and *nos*Z2, VIMSS583543 and VIMSS583547) are adjacent to two cytochrome c553s, a ferredoxin, and a transport accessory protein, and are uniquely embedded within a histidine kinase/response regulator cluster and include *nosD *and a *napGH*-like pair that potentially couples quinone oxidation to cytochrome c reduction. This indicates the NO response might be involved in cell signaling and as a possible general detoxification mechanism for nitric oxide.

The Epsilonproteobacteria *Wolinella succinogenes *is quite similar to *D. aromatica *for nitric oxide reductase genes (both have two *nosZ *genes, a *nosD *gene and a *napGH *pair in the same order and orientation [83]), but the *W. succinogenes *genome lacks the embedded signaling protein cluster. Further, nitric oxide reductase homologs NorDQEBC (VIMSS582097, 582100–582103), along with the cytochrome c' protein (VIMSS582015), which has been shown to bind nitric oxide (NO) prior to its reduction [79], are all present, and potentially act in detoxification roles. It has been shown that formation of anaerobic biofilms of *P. aeruginosa *(which cause chronic lung infections in cystic fibrosis) require NO reductase when quorum has been reached [84], so a role in signaling and complex cell behavior is possible.

*W*. *succinogenes *shares other genome features with *D. aromatica*. It encodes only 2042 orfs, yet has a large number of signaling proteins, histidine kinases, and GGDEF proteins relative to its genome size. It also encodes *nif *genes, several genes similar to virulence factors, and similarity in the nitrous oxide enzyme cluster noted above. *W*. *succinogenes *is evolutionarily related to two pathogenic species (*Helicobacter pylori *and *Campylobacter jejuni*), and displays eukaryotic host interactions, yet is not known to be pathogenic [85]. The distinction between effector molecules causing a pathogenic interaction and a symbiotic one is unclear.

#### Nitrogen Fixation

Nitrogen fixation capability in *D. aromatica *is indicated by a complex of *nif*-like genes (see Table 4), that include putative nitrogenase alpha (NifD, VIMSS583693) and beta (NifK, VIMSS583694) subunits of the molybdenum-iron protein, an ATP-binding iron-sulfur protein (NifH, VIMSS583692), and the regulatory protein NifL (VIMSS583623), that share significant sequence similarity and synteny to the free-living soil microbe *Azotobacter vinelandii*. *D. aromatica *further encodes a complex that is likely to transport electrons to the nitrogenase, by using a six subunit *rnf*ABCDGE-like cluster (VIMSS583616-583619, 583621 and 583622) that is phylogenomically related to the *Rhodobacter capsulatus *complex used for nitrogen fixation [86]. There is a second *rnf*-like NADH oxidoreductase complex composed of VIMSS583911-583916, of unknown involvement (see Fig. 4). *A. aromaticum *EbN1 and *Azoarcus *BH72 each encode two *rnf*-like clusters as well.

Embedded in the putative nitrogen fixation cluster are two gene families involved in urea metabolism (Table 4). This includes the urea transport proteins (UrtABCDE) and urea hydrolase enzyme family (Ure protein family).

#### Hydrogenases associated with nitrogen fixation

Uptake hydrogenase is involved in the nitrogen fixation cycle in root nodule symbionts where it is thought to increase efficiency via oxidation of the co-produced hydrogen (H_2_) [87]. *D. aromatica *encodes a cluster of 13 predicted orfs (Hydrogenase-1 cluster, VIMSS581358-581370; Table 5) that includes a hydrogenase cluster syntenic to the *hox*KGZMLOQR(T)V genes found in *Azotobacter vinelandii*, which reversibly oxidize H_2 _in that organism [88]. This cluster is followed by a second hydrogenase (Hydrogenase-2 cluster, VIMSS581373-581383). The hydrogenase assembly proteins, *hyp*ABF and CDE are included (VIMSS581368-581370 and 581380-581381, and VIMSS3337851 (formerly 581382)) as well as proteins related to the hydrogen uptake (*hup*) genes of various rhizobial microbes [87]. The second region, with the *hyp *and *hyd*-like clusters, lacks overall synteny to any one genome currently sequenced. It does, however, display regions of genes that share synteny with *Rhodoferax ferrireducens*, which displays the highest percent identity across the cluster, both in terms of synteny and protein identity.

**Table 5:** Hydrogenase clusters associated with nitrogen fixation.

VIMSS id	Orthologs	Putative function	Size, aas
581358	HoxK/HyaA/HupS	hydrogenase-1 small subunit	363
581359	HoxG/HyaB/HupL	hydrogenase-1, nickel-dependent, large subunit	598
581360	HoxZ/HyaC/HupC	Ni/Fe-hydrogenase 1 b-type cytochrome subunit	234
581361	HoxM/HyaD/HupD	hydrogenase expression/formation protein	204
581362	HoxL/HypC/HupF	hydrogenase assembly chaperone	100
581363	HoxO/HyaE/HupG	hydrogenase-1 expression	152
581364	HoxQ/HyaF/HupH	nickel incorporation into hydrogenase-1 proteins	287
581365	HoxR/HupI	rubredoxin-type Fe(Cys)4 protein	66
581366	HupJ/(similar to HoxT)	hydrogenase accessory protein	156
581367	HoxV/HupV	membrane-bound hydrogenase accessory protein	308
581368	HypA	hydrogenase nickel insertion protein	113
581369	HypB	hydrogenase accessory factor Ni(2+)-binding GTPase	352
581370	HypF	hydrogenase maturation protein	763
581371	ABC protein	periplasmic component, ABC transporter	260
581372	GGDEF domain	signal transduction, GGDEF	523
581373	Hyb0	hydrogenase-2 small subunit	394
581374	HybA	Fe-S-cluster-containing hydrogenase component	351
581375	HybB	cytochrome Ni/Fe component of hydrogenase-2	386
581376	HybC/HynA	hydrogenase-2 large subunit	570
581377	HybD/HynC	Ni, Fe-hydrogenase maturation factor	159
581378	HupF/HypC	hydrogenase assembly chaperone	96
581379	HybE/HupJ	hydrogenase accessory protein	183
581380	HypC	hydrogenase maturation protein	81
581381	HypD	hydrogenase maturation protein	374
581382/3337851	HypE	hydrogenase maturation protein	330
581383	HoxX/HypX	formation of active hydrogenase	558
581384	HoxA	response regulator with CheY domain (signal transduction)	495
581385	HoxB/HupU	regulatory [NiFe] Hydrogenase small subunit (sensor)	333
581386	HoxC/HupV	regulatory [NiFe] Hydrogenase large subunit (sensor)	472
581397	HupT	histidine kinase with PAS domain sensor	448
581398	HoxN/HupN/NixA	nickel transporter	269

A large region of putative hydrogenases associated with nitrogen fixation is noted. Hydrogenase-1 is composed of *hox/hup *genes, hydrogenase-2 of the *hyb *genes, and the nickel insertion/maturation complex, *hyp *(present in two clusters: *hyp*ABF and *hyp*CDE).

VIMSS581384 encodes a homolog of the HoxA hydrogenase transcriptional regulator, which has been shown to be expressed only during symbiosis in some species [89]. Regulation is indicated by homologs of NtrX (VIMSS581123) and NtrY (VIMSS581124); the NtrXY pathway comprises a two-component signaling system involved in the regulation of nitrogen fixation in *Azorhizobium caulinodans *ORS571 [90].

#### Carbon Fixation via the Calvin-Benson-Bassham cycle

The genes indicative of carbon fixation, using the Calvin cycle, are present in the *D. aromatica *genome. This includes Ribulose 1,5-bisphosphate carboxylase (RuBisCo, VIMSS581681), phosphoribulokinase (cbbP/PrkB, VIMSS581690), and a fructose bisphosphate (fba, VIMSS581693) of the Calvin cycle subtype. The RuBisCo *cbbM *gene is of the fairly rare type II form. *D. aromatica *CbbM displays a surprisingly high 77% amino acid identity to CbbM found in the deep-sea tube worm *Riftia pachyptila *symbiont [91]. In a recent study of aquatic sediments, *Rhodoferax fermentans, Rhodospirillum fulvum *and *R. rubrum *were also found to possess the *cbb*M type II isoform of RuBisCo [92]; this sub-type is shared by a only a few microbial species.

Further putative Cbb proteins are encoded by VIMSS581680 & 581688, candidates for CbbR (regulator for the cbb operon) and CbbY (found downstream of RuBisCo in *R. sphaeroides *[93]), respectively.

The presence of the *cbb*M gene suggests the ability to carry out the energetically costly fixation of CO_2_, though such functionality has yet to be observed, and carbon dioxide fixation capability has been found in only a few members of the microbial community.

There is a potential glycolate salvage pathway indicated by the presence of two isoforms of phosphoglycolate phosphatase (*gph*, VIMSS583850 and 581830). In other organisms, phosphoglycolate results from the oxidase activity of RuBisCo in the Calvin cycle, when concentrations of carbon dioxide are low relative to oxygen. In *Ralstonia (Alcaligenes) eutropha *and *Rhodobacter sphaeroides*, the gph gene (*cbbZ*) is located on an operon along with other Calvin cycle enzymes, including RuBisCo. In *D. aromatica*, the *gph *candidates for this gene (VIMSS583850 and 581830), are removed from the other *cbb *genes on the chromosome in *D. aromatica*; however VIMSS581830 is adjacent to a homolog of Ribulose-phosphate 3-epimerase (VIMSS581829, *rpe*).

The *cco*SNOQP gene cluster codes for a cbb-type cytochrome oxidase that functions as the terminal electron donor to O_2 _in the aerobic respiration of *Rhodobacter capsulatus *[94]. These genes are present in a cluster as VIMSS580484-580486 and VIMSS584273-584274; note that these genes are present in a large number of Betaproteobacteria.

Other carbon cycles, such at the reverse TCA cycle and the Wood-Ljungdahl pathways, are missing critical enzymes in this genome, and are not present as such.

#### Sulfur

Sulfate and thiosulfate transport appear to be encoded in the gene cluster composed of an OmpA type protein (VIMSS581631) followed by orthologs of a sulfate/thiosulfate specific binding protein Sbp (VIMSS581632), a CysU or T sulfate/thiosulfate transport system permease T protein (VIMSS581633), a CysW ABC-type sulfate transport system permease component (VIMSS581634), and a CysA ATP-binding component of sulfate permease (VIMSS581635).

In addition, candidates for the transcriptional regulator of sulfur assimilation from sulfate are present and include: CysB, CysH, and CysI (VIMSS582364, 582360 and 582362, respectively).

**Figure 5:**
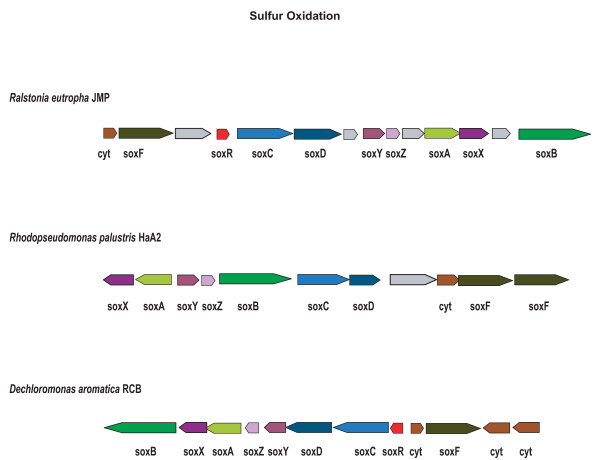
**Sulfur oxidation (thiosulfate to sulfate) candidates in *R. eutropha*, *R. palustris*, and *D. aromatica***. Proposed model for this periplasmic complex is as follows: SoxXA, oxidatively links thiosulfate to SoxY; SoxB, potential sulfate thiohydrolase, interacts with SoxYZ (hydrolyzes sulfate from SoxY to regenerate); SoxCD, a sulfur dehydrogenase; oxidizes persulfide on SoxY to cysteine-S-sulfate and potentially yields 6 electrons per sulphate; SoxC, sulfite oxidase/dehydrogenase with homology to nitrate reductase, induced by thiosulfate; SoxDE, both c-type cytochromes with two heme-type binding sites; and SoxF, a FAD flavoprotein with sulfide dehydrogenase activity. Cyt, cytochrome.

A probable sulfur oxidation enzyme cluster is present and contains homologs of SoxFRCDYZAXB [95], with a putative SoxCD sulfur dehydrogenase, SoxF sulfide dehydrogenase, and SoxB sulfate thiohydrolase, which is predicted to support thiosulfate oxidation to sulphate (see Fig. 5). Functional predictions are taken from Friedrich et al. [95] [see Additional file 7]. A syntenic *sox *gene cluster is also found in *Anaeromyxobacter dehalogens *(although it lacks *sox*FR) and *Ralstonia eutropha*, but not in *A. aromaticum *EbN1. Thiosulfate oxidation, however, has not been reported under laboratory conditions tested thus far, and experimental support for this physiological capability awaits further investigation.

Conversely, the cytoplasmic SorAB complex [96] is not present in *D. aromatica *nor *A. aromaticum *EbN1, although it is found in several other Betaproteobacteria, including *R. metallidurans, R. eutropha*, *R. solanacearum*, *C. violaceum*, and *B. japonicum*.

#### Gene Family Expansion

To determine candidates for recent gene duplication events, extensive phylogenomic profile analyses were conducted for all sets of paralogs in the genome. Flower Power recruitment and clustering against the non-redundant Genbank protein set was done, and the resulting alignments were analyzed using the tree-building SCI-PHY or Belvu based neighbor-joining utilities. The alignment of two or more *D. aromatica *protein sequences in a clade such that they displayed higher % identity to each other than to orthologs present in other species was interpreted as an indication of a probable recent duplication event, either in the *D. aromatica *genome itself or in a progenitor species. Results of this analysis are shown in Table 3.

Potential gene family expansion is indicated in several functional groups, including the following: signaling proteins (including cAMP signaling, histidine kinases, and others), Mhp-like aromatic oxidation complexes, nitrogen metabolism proteins and transport proteins.

Most duplications indicate that a single gene, rather than sets of genes, were replicated. An exception is the Tra/Type IV transport cluster (VIMSS582581-582601 and VIMSS582864-582884) noted previously. In the protein sets for the histidine kinase/response regulator, duplication of histidine kinase appears to occur without duplication of the adjacent response regulator. The paralogs created by recent duplication events are typically found well-removed from one another on the chromosome, although some tandem repeats of single genes were noted. However, the highest percent identity was not found between pairs of genes in tandem repeats.

## Discussion

Discussion of results and analyses concerning aromatic degradation, various predicted metabolic cycles, secretion, signaling, quorum-sensing and gene family expansion are included in the relevant sections, above.

## Conclusion

*Dechloromonas aromatica *strain RCB appears to support a highly complex lifestyle which might involve biofilm formation and interaction with a eukaryotic host. It lacks predicted enzyme families for anaerobic aromatic catabolism, though it supports degradation of several aromatic species in the absence of oxygen. The enzymes responsible for this metabolic function remain to be identified and characterized. It also encodes proteins suggestive of the ability to fix nitrogen and CO_2_, as well as thiosulfate oxidation. Converse to aromatic degradation, these enzymatic functionalities have yet to be experimentally demonstrated. In short, this genome was full of surprises.

The utility of TIGRfams and COGs families in these analyses cannot be overstated. New releases of TIGRfams during the course of this analysis provided new insights and identified new functionality (malonate degradation cluster, PEP-Cterm transport and the epsH putative translocon, and urea degradation all were identified in the TIGRfam 7.0 additions). The HMM model building and assessment utilized as the major annotation approach for this study was employed to cover those protein families of interest that are not currently covered by TIGRfams. We utilized K. Sjölander's modelling and analysis tools, which are highly similar to those used to produce TIGRfams models. Overall, the extensive use of HMMs during this analysis allowed high confidence in predicted protein function, as well as certainty that several families of previously characterized anaerobic degradation enzymes for aromatic compounds are not present (eg BssABCD and BcrAB).

## Authors' contributions

AL coordinated and oversaw the assembly of the genome. WSF, HF and GDB did the initial assembly of the genome. KKS conducted genome assembly, sequence finishing and gap closure activities, and created the final assembly. ST provided internal Joint Genome Institute assembly and analysis tools, and support in their use. KK was involved in the semi-automated genome annotation, and provided support for the VIMSS dataset and data lists from that set. KKS conducted all manual annotation work and protein family analyses described here-in. All authors have read and approved the final manuscript.

## Supplementary Material

Additional file 1 **Phylogenomic analysis: Flower Power, SCI PHY and HMM scoring**. The methodologies used for the annotation of predicted proteins in the *D. aromatica *genome, via hidden Markov model generation and assessment, are described in detail. Click here for file

Additional file 2 **Use of HMM pipelines to assign putative function to *D. aromatica *proteins and determine protein cohort**. A flow diagram is used to depict each of the four approaches employed using HMM generation for determining the presence or absence of specific proteins or enzymes in the *D. aromatica *genome, as well as for annotation of the predicted protein set. Click here for file

Additional file 3 ***Dechloromonas aromatica *RCB genome assembly statistics**. Statistics of the finishing process are shown in table format. Click here for file

Additional file 4 **Statistics for open reading frame predictions**. Number of proteins from this genome having TIGRfam annotations (as of TIGRfam release 7.0) is shown. Click here for file

Additional file 5 **Number of predicted proteins with annotated diguanylate cyclase domains (IPR000160) in various genomes**. The highly variable number of GGDEF domains predicted in proteins from a number of microbial species is shown. Click here for file

Additional file 6 **Type VI secretion cluster**. Effector proteins in the IcmF-like Type VI secretion cluster annotated in *D. aromatica *are listed. Click here for file

Additional file 7 **Putative sulfur oxidation (Sox) cluster**. A number of proteins capable of supporting sulfur oxidation in other species have homologs in the *D. aromatica *genome, and are listed. Click here for file
