# Supplementary material for: Metabolic analysis of the soil microbe Dechloromonas aromatica str. RCB: indications of a surprisingly complex life-style and cryptic anaerobic pathways for aromatic degradation
Source: BMC Genomics. 2009 Aug 3;10:351. doi: 10.1186/1471-2164-10-351 (PMC2907700; doi:10.1186/1471-2164-10-351)
Supplement: Additional file 1 — Phylogenomic analysis: Flower Power, SCI PHY and HMM scoring. The methodologies used for the annotation of predicted proteins in the D. aromatica genome, via hidden Markov model generation and assessment, are described in detail. [file 1471-2164-10-351-S1.doc]

# Phylogenomic analysis: Flower Power , SCI PHY and HMM scoring

Phylogenomic profiling was done to derive functional assignments based on near-neighbors or to confirm the absence of a given protein in the *D. aromatica* genome. Four basic approaches were used [see Additional file 2]. In the first, we employed the Flower Power and SCI-PHY (Subfamily Classification in Phylogenomics) utilities from the UCBerkeley web server ([http://phylogenomics.berkeley.edu](http://phylogenomics.berkeley.edu/) [1, 2]) to profile enzymes specifically from the near-neighbor *A. aromaticum* EbN1 for the creation of Hidden Markov Models (HMMs) that could be scored against the *D. aromatica* protein set. This approach allowed us to determine, with extremely high confidence, whether a protein is truly not present in the *D. aromatica* genome, whether or not automatic annotation had identified it correctly. The same basic protocols were employed for all HMM builds, except for the selection of seed sequences. Phylogenetic trees were assessed by comparing included species with experimentally published orthologs, and in some cases, HMMs were scored against the full set of *D. aromatica* proteins (VIMSS annotation set) in order to compare relative probability scores for individual proteins in the genome [2].

The second use of HMM modeling and phylogenomic tree-building was used to create models for proteins of interest, with known function, to determine whether orthologs are present in the *D. aromatica* genome. Protein models were created based on candidates having enzymatic function related to metabolic function for *D. aromatica* (eg benzylsuccinate synthetase), or for proteins that were suggested by clusters of *D. aromatica* predictions displaying pfam, COG, EC or TIGRfam annotations indicating the possibility of pathways established in other organisms (eg the SOX cluster). For aromatic degradation enzymes, the BRENDA database (http://www.brenda.uni-koeln.de/) [3, 4] was used to select protein sequences for enzymes of known function based on experimental evidence. A characterized protein was used to seed a Flower Power HMM-based recruitment of all related sequences from the Genbank non-redundant protein set, requiring an identity to existing sequences ranging from 0.15 to 0.19 (0.18 most typically produced a robust recruitment set while retaining specificity), and requiring global alignment. Species represented in the recruited set were compared to all species characterized as having that enzyme function, as captured in BRENDA, to assess overall coverage of known enzyme candidates. Often, more than one enzyme was found to be responsible for the same enzymatic process. Therefore, if deemed appropriate, a second Flower Power sequence recruitment was conducted using a seed sequence not captured in the first set of homologous proteins, to create a second HMM model and phylogenetic tree profile for the isozymic set. If the HMM model was to be scored against the full set of *D. aromatica* proteins, alignments were edited using the Belvu alignment editing tool [5] using the methods of Brown et al. [6]. The resulting modified alignment was used to create HMMs using the w0.5 build and hmmscore utilities of UCSanta Cruz as employed by K. Sjölander [7-11]. HMMs were then scored against the complete set of predicted proteins from *D. aromatica*.

The third approach was to carry out *de novo* generation of HMMs for protein sequences of interest that were not adequately described by either TIGRfam or COGs models, or for which no models were available. Proteins or enzymes potentially involved in the metabolic pathways or cell processes of particular interest from the *D. aromatica* VIMSS protein set were used as seed sequences for Flower Power recruitment of phylogenetically related proteins in the Genbank non-redundant data set. In a few instances, Flower Power alignments were used as input for the SCI-PHY utility, and then uploaded into PhyloFacts [12, 13]. Subfamilies generated using the SCI-PHY minimum-encoding-cost criteria were viewed in phylogenetic trees, and the functionality of the protein of interest was inferred based on clade (sub-family) membership, ideally from the experimentally supported functionality of other proteins within the same clade [10, 11, 14]. During the course of this study, TIGRfams 7.0 was released, which contained several models that replicated those generated during this study. In all cases where HMM modeling was used, their annotation predictions agreed with ours.

In the fourth approach using HMM models, internal clustering of all *D. aromatica* proteins (using the VIMSS protein set) was employed to create a set of paralogous proteins within the *D. aromatica* genome using an internal suite of computational tools as employed by K. Sjölander, UCBerkeley [1]. Comparison of these sets of full-length paralogs allowed identification of putative enzymes forming sequential steps in a given catabolic pathway, many of which display physical proximity along the chromosome (eg. the Mhp families of proteins). It also allowed identification of smaller subunits in multi-enzyme complexes, which were often missed by the high-throughput annotation pipelines employed.

1. Krishnamurthy N, Brown D, Sjolander K: **FlowerPower: clustering proteins into domain architecture classes for phylogenomic inference of protein function**. *BMC Evol Biol* 2007, **7 Suppl 1**:S12.

2. Brown DP, Krishnamurthy N, Sjolander K: **Automated protein subfamily identification and classification**. *PLoS Comput Biol* 2007, **3**(8):e160.

3. Schomburg I, Chang A, Schomburg D: **BRENDA, enzyme data and metabolic information**. *Nucleic Acids Res* 2002, **30**(1):47-49.

4. Barthelmes J, Ebeling C, Chang A, Schomburg I, Schomburg D: **BRENDA, AMENDA and FRENDA: the enzyme information system in 2007**. *Nucleic Acids Res* 2007, **35**(Database issue):D511-514.

5. Sonnhammer EL, Hollich V: **Scoredist: a simple and robust protein sequence distance estimator**. *BMC Bioinformatics* 2005, **6**:108.

6. Brown D, Krishnamurthy N, Dale JM, Christopher W, Sjolander K: **Subfamily hmms in functional genomics**. *Pac Symp Biocomput* 2005:322-333.

7. Hughey R, Krogh A: **Hidden Markov models for sequence analysis: extension and analysis of the basic method**. *Comput Appl Biosci* 1996, **12**(2):95-107.

8. Karplus K, Karchin R, Barrett C, Tu S, Cline M, Diekhans M, Grate L, Casper J, Hughey R: **What is the value added by human intervention in protein structure prediction?** *Proteins* 2001, **Suppl 5**:86-91.

9. Krogh A, Brown M, Mian IS, Sjolander K, Haussler D: **Hidden Markov models in computational biology. Applications to protein modeling**. *J Mol Biol* 1994, **235**(5):1501-1531.

10. Brown D, Sjolander K: **Functional classification using phylogenomic inference**. *PLoS Comput Biol* 2006, **2**(6):e77.

11. Sjolander K: **Phylogenomic inference of protein molecular function: advances and challenges**. *Bioinformatics* 2004, **20**(2):170-179.

12. Glanville JG, Kirshner D, Krishnamurthy N, Sjolander K: **Berkeley Phylogenomics Group web servers: resources for structural phylogenomic analysis**. *Nucleic Acids Res* 2007, **35**(Web Server issue):W27-32.

13. Krishnamurthy N, Brown DP, Kirshner D, Sjolander K: **PhyloFacts: an online structural phylogenomic encyclopedia for protein functional and structural classification**. *Genome Biol* 2006, **7**(9):R83.

14. Zmasek CM, Eddy SR: **RIO: analyzing proteomes by automated phylogenomics using resampled inference of orthologs**. *BMC Bioinformatics* 2002, **3**:14.
