# Supplementary material for: Metabolic analysis of the soil microbe Dechloromonas aromatica str. RCB: indications of a surprisingly complex life-style and cryptic anaerobic pathways for aromatic degradation
Source: BMC Genomics. 2009 Aug 3;10:351. doi: 10.1186/1471-2164-10-351 (PMC2907700; doi:10.1186/1471-2164-10-351)
Supplement: Additional file 2 — Use of HMM pipelines to assign putative function to D. aromatica proteins and determine protein cohort. A flow diagram is used to depict each of the four approaches employed using HMM generation for determining the presence or absence of specific proteins or enzymes in the D. aromatica genome, as well as for annotation of the predicted protein set. [file 1471-2164-10-351-S2.doc]

## Use of HMM pipelines to assign putative function to D. aromatica proteins and determine protein cohort.

BRENDA database

Seed sequence - enzyme of known function

Azoarcus EbN1 protein set (VIMSS, Genbank)

Seed sequence - enzyme of known function

Assess recruited species against BRENDA list

Build HMM; score against *D. aromatica* protein set

If indicated, create second HMM model using different seed sequence from non-recruited species

Determine whether *D. aromatica* protein(s) are recruited to HMM

Build HMM; score against *D. aromatica* protein set

**A.**

**B.**

*D. Aromatica* protein set (VIMSS)

*D. Aromatica* protein set (VIMSS)

Seed sequence - protein of interest

Assess tree, alignments of recruited protein set

Species assessment – subfamily members vs phylogenomic profile

Functional analysis –identify proteins having experimental evidence

Enzyme/ protein paralogous groups

Make functional prediction for *D. aromatica* protein

Internal clustering using global alignment

Pathway clusters aggregated and assessed

**C.**

**D.**

A. In order to determine the presence or absence of enzymes of known function in the *D. aromatica* genome, enzymes of known function from the BRENDA database were used to seed HMM models that were then scored against the *D. aromatica* protein set. B. Determining the presence or absence of enzymes of known function specifically from the *Azoarcus* EbN1 genome, using the same protocol as (A). C. Proteins of interest from the *D. aromatica* genome were used as seed sequences in HMM recruitment schema to determine putative function. Functional predictions were based on experimental evidence (if present) for proteins recruited to the same clade in phylogenomic tree profiles. D. *D. aromatica* proteins were internally clustered to determine paralogous proteins within the genome, for further analysis. The resulting protein cluster set was also employed for identifying additional members in multi-component protein pathways (eg the Mhp protein families).
