# Supplementary material for: Metabolic analysis of the soil microbe Dechloromonas aromatica str. RCB: indications of a surprisingly complex life-style and cryptic anaerobic pathways for aromatic degradation
Source: BMC Genomics. 2009 Aug 3;10:351. doi: 10.1186/1471-2164-10-351 (PMC2907700; doi:10.1186/1471-2164-10-351)
Supplement: Additional file 3 — Dechloromonas aromatica RCB genome assembly statistics. Statistics of the finishing process are shown in table format. [file 1471-2164-10-351-S3.doc]

## *Dechloromonas aromatica* RCB genome assembly statistics.

| **Genome Statistics** | **Number** |
| --- | --- |
| **Genome length (bp)** | **4,501,104** |
| **Plasmids** | **none** |
| **Depth of Coverage** | **24** |
| **Reads:** |  |
| **40kb** | **4,399** |
| **8kb*** | **64,680** |
| **3kb*** | **61,325** |
| **PCR** | **196** |
| **Total used in final assembly** | **130,600** |
|  |  |
| ***includes primer-walking reads for finishing** |  |
|  |  |
| **5s rRNA** | **4** |
| **16s rRNA** | **4** |
| **23s rRNA** | **4** |
|  |  |
| **Total number of predicted orfs:** |  |
| **VIMSS** | **4,170** |
| **JGI** | **4,204** |

Statistics for finishing the *D. aromatica* genome, and resulting annotation, are shown.
