# Supplementary material for: Metabolic analysis of the soil microbe Dechloromonas aromatica str. RCB: indications of a surprisingly complex life-style and cryptic anaerobic pathways for aromatic degradation
Source: BMC Genomics. 2009 Aug 3;10:351. doi: 10.1186/1471-2164-10-351 (PMC2907700; doi:10.1186/1471-2164-10-351)
Supplement: Additional file 4 — Statistics for open reading frame predictions. Number of proteins from this genome having TIGRfam annotations (as of TIGRfam release 7.0) is shown. [file 1471-2164-10-351-S4.doc]

## Statistics for open reading frame predictions.

| **VIMSS** | **Number** | **Percent** |
| --- | --- | --- |
| **Total Number of Predicted Proteins (orfs)** | **4170** | **100%** |
|  |  |  |
| **TIGRfams:** |  |  |
| **Categorized by TIGRfam (total: domain, hypothetical, equivalog)** | **1368** | **33%** |
| **Categorized by TIGRfam equivalog** | **723** | **18%** |
| **Number of predicted orfs without any type of TIGRfam coverage** | **2802** | **67%** |
|  |  |  |
| **Number of predicted orfs with no annotation** | **539** | **13%** |

Statistics for predicted coding sequences (orfs) in the VIMSS database. Number of predicted orfs with no annotation enumerates all predictions with no domain, ortholog, COG, TIGRFAM, InterPro or any other homologies to support functional predictions.
