# Supplementary material for: Metabolic analysis of the soil microbe Dechloromonas aromatica str. RCB: indications of a surprisingly complex life-style and cryptic anaerobic pathways for aromatic degradation
Source: BMC Genomics. 2009 Aug 3;10:351. doi: 10.1186/1471-2164-10-351 (PMC2907700; doi:10.1186/1471-2164-10-351)
Supplement: Additional file 5 — Number of predicted proteins with annotated diguanylate cyclase domains (IPR000160) in various genomes. The highly variable number of GGDEF domains predicted in proteins from a number of microbial species is shown. [file 1471-2164-10-351-S5.doc]

## Number of predicted proteins with annotated diguanylate cyclase domains (IPR000160) in various genomes*.*

| **Genome** | **IPR000160, GGDEF domain** |
| --- | --- |
| ***Desulfuromonas spp.*** | **109** |
| ***Shewanella sediminis* HAW-EB3** | **71** |
| ***Hahella chejuensis* KCTC 2396** | **65** |
| ***Dechloromonas aromatica* RCB** | **57** |
| ***Alteromonadales bacterium* TW-7** | **55** |
| ***Shewanella oneidensis* MR-1** | **54** |
| ***Azoarcus* BH72** | **51** |
| ***Magnetospirillum magneticum* AMB-1** | **46** |
| ***Chromobacterium violaceum* ATCC 12472** | **43** |
| ***Pseudomonas fluorescens* Pf-5** | **40** |
| ***Bradyrhizobium japonicum*** | **35** |
| ***Ralstonia eutropha* JMP134** | **28** |
| ***Aromatoleum aromaticum* (formerly *Azoarcus*) EbN1** | **26** |
| ***Ralstonia solanacearum*** | **23** |
| ***Nostoc punctiforme*** | **22** |
| ***Escherichia coli* K12** | **19** |
| ***Rhodobacter sphaeroides* 2.4.1** | **16** |
| ***Prochlorococcus*** | **0** |

Total number of proteins annotated with a GDDEF domain as quantified in the VIMSS database.
