# Supplementary material for: Metabolic analysis of the soil microbe Dechloromonas aromatica str. RCB: indications of a surprisingly complex life-style and cryptic anaerobic pathways for aromatic degradation
Source: BMC Genomics. 2009 Aug 3;10:351. doi: 10.1186/1471-2164-10-351 (PMC2907700; doi:10.1186/1471-2164-10-351)
Supplement: Additional file 6 — Type VI secretion cluster. Effector proteins in the IcmF-like Type VI secretion cluster annotated in D. aromatica are listed. [file 1471-2164-10-351-S6.doc]

## Type VI secretion cluster.

| **VIMSS id** | **TIGRfam** | **TIGRfam description** | **Size, aas** |
| --- | --- | --- | --- |
| **582995** | **TIGR03348** | **IcmF-like, homologs associated with type VI secretion systems** | **1270** |
| **582996** | **TIGR03373** | **Found exclusively in type VI secretion-associated gene clusters** | **327** |
| **582997** | **-** | **OmpA/MotB-like protein** | **261** |
| **582998** | **TIGR03363** | **Chp8 Type VI secretion systems containing an ImpA-related N-terminal domain** | **365** |
| **582999** | **TIGR01646 & TIGR03361** | **Rhs element Vgr protein** | **911** |
| **583000** | **TIGR03349** | **Type VI secretion systems, IcmF family** | **257** |
| **583001** | **TIGR03353** | **Chp4, associated with type VI secretion loci** | **447** |
| **583002** | **TIGR03352** | **IAHP, associated with type VI secretion loci** | **189** |
| **583003** | **TIGR03558** | **Chp5, Evp-like (Edwardella virulence protein)** | **170** |
| **583004** | **TIGR03555** | **Chp2, Evp-like (Edwardella virulence protein)** | **494** |
| **583005** | **TIGR03344** | **Hcp hemolysin co-regulated protein; exported, homohexameric ring-forming virulence protein** | **178** |
| **583006** | **TIGR03357** | **All members associated with type VI secretion loci. Similar to acidic lysozyme activity in some phages** | **159** |
| **583007** | **TIGR03359** | **Chp6, associated with type VI secretion. Mutation is associated with impaired virulence, such as impaired infection of plants.** | **610** |
| **583008** | **TIGR03347** | **IAHP-related loci, type VI secretion system** | **354** |
| **583009** | **TIGR03345** | **ClpV1, related to chaperone ClpB - ATPase function only** | **899** |
| **583011** | **TIGR01646 & TIGR03361** | **VgrP Type VI secretion Rhs element, found in Rhs classes G and E** | **933** |

The type VI secretion locus is shown, with brief TIGRfam family description. Chp: conserved hypothetical protein. Type VI secretion is a fairly recently described secreton occurring in gram negative bacteria. It was initially associated with pathogenicity, but has since been found to be involved in host interactions, and not restricted to pathogens.
