# Supplementary material for: Metabolic analysis of the soil microbe Dechloromonas aromatica str. RCB: indications of a surprisingly complex life-style and cryptic anaerobic pathways for aromatic degradation
Source: BMC Genomics. 2009 Aug 3;10:351. doi: 10.1186/1471-2164-10-351 (PMC2907700; doi:10.1186/1471-2164-10-351)
Supplement: Additional file 7 — Putative sulfur oxidation (Sox) cluster. A number of proteins capable of supporting sulfur oxidation in other species have homologs in the D. aromatica genome, and are listed. [file 1471-2164-10-351-S7.doc]

## Putative sulfur oxidation (sox) cluster.

| **VIMSS id** | **Ortholog** | **Putative function** | **Size, aas** |
| --- | --- | --- | --- |
| **582151** | **cytochrome c553** | **cytochrome-c oxidase (EC1.9.3.1)** | **200** |
| **582152** | **cytochrome c553** | **cytochrome-c oxidase (EC1.9.3.1)** | **205** |
| **582153** | **SoxF** | **FAD-dependent sulfide dehydrogenase** | **425** |
| **582154** | **cytochrome c553** | **cytochrome-c oxidase (EC1.9.3.1)** | **101** |
| **582155** | **transcriptional regulator** | **DNA binding regulatory protein (ArsR family)** | **106** |
| **582156** | **SoxC** | **sulfite oxidase (EC1.8.3.1)** | **448** |
| **582157** | **SoxD** | **cytochrome c551/c552** | **348** |
| **582158** | **SoxY** | **thiosulfate acceptor** | **155** |
| **582159** | **SoxZ** | **thiosulfate acceptor subunit** | **103** |
| **582160** | **SoxA** | **cytochrome c heme-binding** | **271** |
| **582161** | **SoxX** | **cytochrome c, monohaem** | **215** |
| **582162** | **SoxB** | **thiohydrolase, regenerates soxY** | **573** |

Potential sulfur oxidation cluster (*sox* genes) as annotated in the *D. aromatica* genome.
